# Serum metabolic and minerals profile in norgestomet primed postpartum anestrous surti buffaloes

**DOI:** 10.14202/vetworld.2015.625-630

**Published:** 2015-05-21

**Authors:** Sanjay C. Parmar, C. T. Khasatiya, J. K. Chaudhary, R. V. Patel, H. B. Dhamsaniya

**Affiliations:** 1 Department of Animal Reproduction, Gynaecology and Obstetrics, Anand Agricultural University, Anand, Gujarat, India; 2 Department of Veterinary Gynaecology and Obstetrics, Navsari Agricultural University, Navsari, Gujarat, India; 3 Sumul Animal Breeding Centre, Surat, Gujarat, India; 4 Sabarmati Ashram Gaushala, Bidaj Farm, Kheda, Gujarat, India

**Keywords:** anestrous, buffaloes, cholesterol, micro-minerals, norgestomet, pregnant mare serum gonadotropin, protein

## Abstract

**Aim:**

The study was undertaken to find out the serum metabolic and minerals profile in postpartum anestrous surti buffaloes treated with norgestomet ear implants alone and in combination with pregnant mare serum gonadotropin (PMSG).

**Materials and Methods:**

The study was conducted on 18 postpartum anestrous Surti buffaloes divided into three groups of six animals each at random to conduct the experiment. The buffaloes in Group-I and Group-II were implanted with Crestar ear implant for 9 days together with 2 ml injection of Crestar solution given i/m on the day of the implant insertion. In Group-II, additionally 500 IU PMSG was given i/m on the day of implant removal, whereas the buffaloes in Group-III served as anestrous control group and received 5 ml Normal Saline i/m on day 0 and 9 as a placebo treatment.

**Results:**

The overall serum total protein values did not differ significantly (p > 0.05) between time (days) intervals in any of the groups. The mean serum total cholesterol levels at 10^th^ day and on the day of estrus were found significantly lower (p < 0.05) in the control group as compared to treatment Groups I and II. However, there was no significant difference (p > 0.05) at 10^th^ day and on the day of estrus between treatment groups (T1 and T2). The overall mean serum cobalt, zinc, iron, and manganese values did not differ significantly (p > 0.05) between different time intervals among any of the groups, except copper which was significantly lower (p < 0.05) at 10^th^ day in control group as compared to treatment groups.

**Conclusion:**

Microelements cannot be synthesized in the body. Hence, it is concluded that the mineral mixture should be supplied daily in the animals ration to suffice the requirement of the trace elements. The mean serum metabolic and micro-minerals profiles in treatment and control groups revealed that overall mean serum total protein, cholesterol, copper, and zinc levels were apparently higher in treatment groups whereas, mean serum cobalt, iron, and manganese concentration had no consistent trend between treatment and control groups of Surti buffaloes.

## Introduction

The postpartum period plays a pivotal role in bovine reproduction. The duration of postpartum anestrous has an important influence on reproductive performance. There are certain biochemical parameters, which directly influence the reproductive performance of animals either through stimulating hormone synthesis, hormone action or response of the target tissue by acting as precursor for hormone synthesis. Nutritional factors needed for successful reproduction are the same as those needed for maintenance, growth and lactation, that includes protein, energy, mineral and vitamins, and deficiency or excess, any of these components, which is serious enough to affect reproduction will also affect other physiological functions. Lack of minerals and trace elements such as copper, cobalt, manganese, zinc, etc., upset the proper functioning of the genital organs [1]. Trace elements may function as cofactors, as activators of enzymes or stabilizers of secondary molecular structure [2]. Ruminants frequently are subjected to severe dietary deficiencies of trace elements such as copper, cobalt, selenium, iodine, manganese, and zinc. Concomitant infertility in cattle is believed to be associated with enzymatic dysfunctions resulting from these deficiencies [3].

Optimum protein level is necessary for the development of endocrine and sex organs. The ill effect of low protein on reproduction is through pituitary and sex glands. Protein deficiency retards the development of reproductive organs and was considered to be a factor responsible for failure or delay in onset of postpartum estrus [4]. Cholesterol is synthesized from acetate with a series of intermediate substances. It is an essential precursor for steroid hormones of testis, ovary and adrenal cortex [5]. Various minerals are the essential nutrients bearing a significant role in the reproductive performance of ruminants. Deficiency or excess of minerals like Co, Cu and Zn have been associated with subnormal fertility and anoestrous conditions.

Hence, the aim of the present investigation was carried out study the serum metabolic and minerals profile in postpartum anestrous Surti buffaloes treated with Norgestomet ear implants alone or in combination with pregnant mare serum gonadotropin (PMSG).

## Materials and Methods

### Ethical approval

The study was approved by the University Animal Ethics Committee of Navsari Agricultural University constituted for research purpose.

### Animals

The study was conducted on 18 anestrous (inactive ovaries) Surti buffaloes from 45 to 120 days postpartum maintained at University farm, Navsari, Gujarat between November, 2013 and April, 2014. All these buffaloes had normal calving and subsequent normal genital health as assessed gyneco-clinically. Estrus occurrence was detected daily in all the buffaloes with the help of teaser bull parading in morning and evening hours. The animals which were not exhibiting overt signs of estrus during routine heat detection program were segregated and subjected to rectal palpation. The animals with smooth inactive ovaries (no palpable follicle or corpus luteum) on twice per rectal palpation 11 days apart were considered as postpartum anestrous buffaloes. Thereafter, the buffaloes randomly divided into 3 equal groups each comprising 6 buffaloes.

### Grouping of experimental animals

Total eighteen postpartum anestrous Surti buffaloes divided into three groups of six animals each at randomly selected for the experiment. All the buffaloes were in second to third parity and four to 6-year-old. The buffaloes in Group-I and Group-II were implanted with siliastic Crestar ear implant (3.3 mg Norgestomet, Intervet International B.V. Boxmeer, Netherlands) subcutaneously in the middle of the outer surface of the ear pinnae with the help of special applicator along with i/m injection of 2 ml Crestar solution containing 3 mg Norgestomet and 5 mg estradiol valerate and considered as day 0. On day 9, the implants were removed from all the buffaloes. Buffaloes in Group-II also received additional Injection of Folligon 500 IU (Pregnant Mare Serum Gonadotrophin, Intervet) on day 9, immediately after implant removal, while buffaloes in Group-III served as control and were given 5 ml Normal Saline i/m as placebo treatment on days 0 and 9. All the experimental animals were maintained under uniform manage mental conditions and experiment was approved by the animal Ethics Committee and University.

### Blood collection

Approximately 10 ml blood samples were collected in the vaccutainers without anticoagulant from all the selected buffaloes on days 0 (prior to treatment), 5 (during treatment), 10 (after treatment) and on day of estrus by jugular vein puncture. The serum was separated out after clotting of blood by centrifugation at 3000 rpm for 15 min and stored at −20°C in a deep freezer until analyzed.

### Metabolic and micro-minerals estimation

Estimation of total protein was done by Biuret method and that of total cholesterol as per Enzymatic Endpoint method using assay kits and procedure of Randox Laboratories Ltd., UK, on an autoanalyzer (Merck’s Micro-lab 300 analyzer, Vital Scientific, DIEREN-Netherlands). The levels of trace minerals, *viz*. copper, cobalt, zinc, iron, and manganese were determined according to the method of Krishna and Ranjhan [6]. The blood serum samples (0.5 ml each) were wet digested with 4.5 ml volume of the tri-acid mixture (perchloric acid: sulphuric acid: nitric acid; 1:2:1) on a hot plate. The clear transparent residues were diluted in double glass-distilled water and the final volume was made to 25 ml. These aliquots were then used for estimation of trace elements on an Atomic Absorption Spectrophotometer.

### Statistical analysis

The data collected were suitably tabulated and analyzed following standard statistical methods of ANOVA and Duncan’s new multiple range test as shown by Steel and Torrie [7].

## Results and Discussion

### Total protein

The mean serum total protein levels of anestrous buffaloes did not differ significantly (p > 0.05) within and between the treatment and control groups at any time (days) intervals including overall mean values. The present overall mean serum total protein concentration was in agreement with the report of Soni [8] in postpartum anestrous Surti buffaloes. The non-significant effect of periods was in agreement with the Chaudhari [9] in the Kankrej heifers with Norgestomet treatment and Dhami *et al*. [10] in the Surti buffaloes with GnRH/PG treatment. Khasatiya *et al*. [11] reported significantly higher overall pooled mean of blood plasma total protein in postpartum anestrous treatment group than that of control group (7.70±0.13 vs. 7.01±0.21 g/dl), in Surti buffaloes. Serum protein levels change with different stages of reproduction, depending upon the feed intake of the animal. Optimum protein level is necessary for the development of endocrine glands and sex organs. The ill effect of low protein on reproduction is through pituitary and sex glands. Protein deficiency retarded the development of reproductive organs and was considered to be a factor responsible for failure or delay in onset of postpartum estrus [12] (Table-1).

**Table-1:** Serum total protein (g/dl) and total cholesterol (mg/dl) levels at different time intervals/days in anestrous treated and control groups of buffaloes (Mean±SEM).

Groups/treatments	0 day (pre-treatment)	5^th^ day (during treatment)	10^th^ day (post-treatment)	Day of estrus	Overall
Total protein					
Group-I	6.76±0.45	6.97±0.46	7.39±0.24	7.86±0.64	7.24±0.24
Group-II	6.93±0.27	7.39±0.24	7.71±0.44	8.00±0.81	7.51±0.24
Group-III	6.66±0.28	6.76±0.45	6.79±0.36	7.27±0.36	6.87±0.18
Total cholesterol					
Group-I	129.97±14.26^a^	132.42±09.72^a^	145.40±10.92^b^	160.65±08.76^b^	142.11±05.77^b^
Group-II	143.87±13.17^a^	149.68±07.68^a^	163.98±01.28^b^	168.48±06.82^b^	156.50±04.43^c^
Group-III	121.42±09.72^a^	122.20±13.31^a^	124.07±01.94^a^	140.26±03.31^a^	126.99±04.26^a^

Group-I=Norgestomet, Group-II=Norgestomet+PMSG, Group-III=Anestrous control, Means bearing different superscripts within a column differ significantly (p<0.05), SEM=Standard error of the mean, PMSG=Pregnant mare serum gonadotropin

### Total cholesterol

The mean serum total cholesterol level between different time intervals within the group did not show a significant difference. However, the mean serum total cholesterol levels at 10^th^ day of treatment and on the day of estrus were significantly lower (p < 0.05) in control Group-III as compared to Group-I and Group-II, which were statistically similar. The increasing trends toward follicular phase may be the reason for significantly higher overall values on the day of estrus as compared to other days in all three groups. These findings on overall mean serum total cholesterol concentrations in anestrous buffaloes are in close agreement with those (122.80±4.85 to 156.63±14.59 mg/dl) reported by earlier workers [11, 13-16] in anestrous cows and buffaloes, and 153.74±5.46 mg/dl reported by Chaudhari [9] in the delayed pubertal Kankrej heifers following norgestomet ear implant plus PMSG treatment. In addition to this, higher overall mean plasma cholesterol levels in Surti buffaloes during the breeding season were recorded by Prajapati [17]. Whereas, compared to present values, very low mean blood serum total cholesterol concentration reported by Ali and Shukla [18] in anestrous buffaloes. Moreover, earlier worker have reported that mean cholesterol level was higher in cyclic buffaloes than those reported in this study of Surti buffaloes that had inactive ovaries [16], Selvaraju and Rajasundaram [14] and Chaudhari [9] reported significant variations in mean serum total cholesterol concentrations before, during and after treatment as well as on the day of estrus in norgestomet alone and norgestomet plus PMSG treatment groups. Though, the normal value of serum total cholesterol is ranging from 100 to 200 mg/dl [19].

In the present study, mean serum total cholesterol levels gradually increased from the day of implant insertion to the day of induced estrus in all the three groups and the values were found highest on the day of estrus particularly in treated groups. This trend was in agreement with Raju *et al*. [13], Selvaraju and Rajasundaram [14] and Chaudhari [9] in cattle probably due to the administration of exogenous estradiol valerate, and/or increased follicular activity and steroidogenesis. Purohit and Kohli [20] opined that the increase in cholesterol level of blood serum at estrus was due to the mechanism by which estrogens affected the complex inter-relationships of pituitary-thyroid-adrenal functions and the estrogens had an effect on the carbohydrate metabolism that in turn caused increased production of cholesterol in endocrine gland tissue from acetate. Steroid hormones have a direct relationship with cholesterol metabolism [21]. The higher cholesterol level in the cycling animals is indicative of more secretion of steroids during estrus due to increased ovarian activity [22]. Kavani *et al*. [23] opined that the low cholesterol level might have resulted in the inadequate synthesis of sex steroid hormones leading to the anestrous condition.

### Copper

The mean serum copper levels at different time intervals revealed non-significant variation (p > 0.05) in all three groups. Moreover, the mean serum copper level at 10^th^ day (post-treatment) in control Group-III was significantly lower (p < 0.05) as compared to treatment Groups I and II. The present overall mean serum copper level was in agreement with the reports of Shah [24] and Khasatiya *et al*. [11] in Surti buffaloes. Chaudhari [9] reported mean serum copper concentrations to vary significantly at different time intervals in the norgestomet treatment group as well as in norgestomet plus PMSG treatment group. Importance of copper in the animals feed stuff has been discussed by various workers as copper levels appear to be influenced by hormones of reproduction, the higher serum copper level indicated higher estrogenic and lower follicle stimulating hormone (FSH) and luteinizing hormone (LH) activity in the serum and its concentration was found to be highest during peak breeding season [25,26]. The critical level of copper was suggested as 0.65 µg/ml by McDowell [27] below which the clinical signs of deficiency may occur (Table-2).

**Table-2:** Serum copper, cobalt, zinc, iron, and manganese levels (µg/ml) at different time intervals/days in anestrous treated and control groups of buffaloes (mean±SEM).

Groups/treatments	0 day (pre-treatment)	5^th^ day (during treatment)	10^th^ day (post treatment)	Day of estrus	Overall
Copper					
Group-I	0.73±0.08_a_^w^	0.78±0.02_a_^w^	0.82±0.02_b_^w^	0.85±0.01_a_^w^	0.80±0.02_b_
Group-II	0.74±0.07_a_^w^	0.79±0.01_a_^w^	0.84±0.01_b_^w^	0.87±0.05_a_^w^	0.81±0.02_b_
Group-III	0.73±0.07_a_^w^	0.74±0.02_a_^w^	0.71±0.02_a_^w^	0.76±0.01_a_^w^	0.74±0.02_a_
Cobalt					
Group-I	0.60±0.10_a_^w^	0.63±0.12_a_^w^	0.61±0.05_a_^w^	0.63±0.02_a_^w^	0.62±0.04_a_
Group-II	0.61±0.07_a_^w^	0.63±0.06_a_^w^	0.64±0.05_a_^w^	0.65±0.11_a_^w^	0.63±0.03_a_
Group-III	0.59±0.04_a_^w^	0.62±0.05_a_^w^	0.61±0.06_a_^w^	0.64±0.07_a_^w^	0.62±0.03_a_
Zinc					
Group-I	1.71±0.11_a_^w^	1.73±0.14_a_^w^	1.74±0.14_a_^w^	1.70±0.10_a_^w^	1.72±0.06_a_
Group-II	1.80±0.23_a_^w^	1.73±0.19_a_^w^	1.76±0.18_a_^w^	1.82±0.19_a_^w^	1.78±0.09_a_
Group-III	1.71±0.13_a_^w^	1.65±0.11_a_^w^	1.63±0.09_a_^w^	1.67±0.09_a_^w^	1.67±0.05_a_
Iron					
Group-I	3.23±0.07_a_^w^	3.26±0.06_a_^w^	3.26±0.03_a_^w^	3.28±0.08_a_^w^	3.26±0.03_a_
Group-II	3.25±0.06_a_^w^	3.25±0.04_a_^w^	3.28±0.06_a_^w^	3.33±0.04_a_^w^	3.28±0.02_a_
Group-III	3.20±0.05_a_^w^	3.22±0.04_a_^w^	3.24±0.02_a_^w^	3.30±0.07_a_^w^	3.24±0.02_a_
Manganese					
Group-I	0.14±0.01_a_^w^	0.14±0.01_a_^w^	0.15±0.01_a_^w^	0.15±0.01_a_^w^	0.15±0.01_a_
Group-II	0.15±0.02_a_^w^	0.15±0.04_a_^w^	0.15±0.01_a_^w^	0.15±0.02_a_^w^	0.15±0.01_a_
Group-III	0.14±0.01_a_^w^	0.15±0.01_a_^w^	0.14±0.01_a_^w^	0.14±0.01_a_^w^	0.14±0.04_a_

Group-I=Norgestomet, Group-II=Norgestomet+PMSG, Group-III=Anestrous control, Means bearing common superscripts (w) within a row and (a, b, c) within a column do not differ significantly (p>0.05), SEM=Standard error of the mean

### Cobalt

In the present study, the mean serum cobalt levels of anestrous Surti buffaloes neither differed significantly between days nor between groups at any of the intervals, and corroborated with Soni [8]. The observations made in the present study before, during and after treatment as well as on the day of estrus were found to be non-significant between and within the group including overall means were in agreement with the findings of Khasatiya *et al*. [11] and Soni [8] in postpartum Surti buffaloes. Cobalt is required for the synthesis of vitamin B12 and its deficiency has been associated with anestrous, abortions, non-functional ovaries [28], birth of weak calves and general infertility [29], delayed onset of puberty [5,30]. The most common manifestation of cobalt deficiency is marked reduction in conception rate with reduction in estrus during the normal breeding season.

### Zinc

The mean serum zinc concentrations of anestrous Surti buffaloes also did not differ significantly (p > 0.05) between sampling days or between groups at any of the days, and showed close agreement with Soni [8]. Chaudhari [9] also reported comparable findings following Norgestomet ear implant alone as well as in combination with PMSG treatment groups in Kankrej heifers. The critical level of zinc has been suggested as 0.6-0.8 µg/ml by McDowell [27], below which the clinical signs of deficiency may occur. A reduction in zinc level might interfere with prostaglandin receptor-mediated phase and consequently the luteolytic process which in turn causes some of the reproductive pathologies [31]. Optimum level of zinc is essential to maintain the activity of FSH and LH [32].

### Iron and manganese

The mean serum iron and manganese levels of anestrous Surti buffaloes also did not differ significantly (p > 0.05) between sampling days or between groups at any of the days, and the findings were in close agreement with Soni [8], Chaudhari [9], and Khasatiya *et al*. [11]. Although, Shah [24] found significantly higher overall pooled me n iron level in anestrous treated than anestrous control Surti buffaloes (1.18±0.03 vs. 1.05±0.04 µg/ml). In addition to this, Maynard and Loosli [33] suggested that iron has little importance in reproduction as compared to copper and zinc. However, low level of iron could possibly result in improper oxygenation of the uterus resulting in impaired nutrition in the uterus for the concept us causing death of the embryo [34]. Manganese levels have been found to be influenced by the progesterone levels during dioestrus. In cycling animals, levels reduce during dioestrus phase in contrast to anestrous animals [35]. It might be due to the major involvement of the manganese ions in the energy-producing ATP requiring reactions, which might be pronounced at the time of stressful events like estrus. Since granulosa cells of the ovary require manganese for the follicular development its deficiency might lead to degeneration of granulosa cells [5].

Kumar *et al*. [36] suggests that either hormonal factors or the trace minerals itself is responsible to activate the enzymes and endocrine systems of the pubertal and sexually matured animals to trigger the activity of hypothalamo-hypophyseal and gonadal system to bring the animal in cyclicity. Trace minerals might have caused the elevation of FSH and FSH: LH ratio resulting into folliculogenesis. The concentrations of macro and micro minerals are perhaps essential for the normal ovulatory processes [37-42]. Improvements in pregnancy rates after mineral mixture supplementation have been reported in Egyptian buffalo [43]. Microelements cannot be synthesized in the body. Hence, it is concluded that the mineral mixture should be supplied daily in the animals ration to suffice the requirement of the trace elements. The mean serum metabolic and micro-minerals profiles in treatment and control groups revealed that overall mean serum total protein, cholesterol, copper, and zinc levels were apparently higher in treatment group whereas, mean serum cobalt, iron, and manganese concentration had no consistent trend between treatment and control groups of Surti buffaloes. No significant differences were observed in the various serum micro-minerals (Co, Zn, Fe, and Mn) constituents between treated and control groups at different time intervals except copper, which might be due to organized farm of Surti buffaloes under study being maintained on optimum nutritional supplementation and healthcare strategies.

## Authors’ Contributions

CTK designed the experiment. SCP carried out the study along with JKC, RVP, and HBD. SCP and CTK analyzed the data and prepared the manuscript. CTK reviewed the manuscript. All authors participated in the scientific discussion. All authors read and approved the final manuscript.
